# Near Fermi Superatom State Stabilized by Surface State Resonances in a Multiporous Molecular Network

**DOI:** 10.1021/acs.nanolett.1c01200

**Published:** 2021-05-26

**Authors:** Shigeki Kawai, Mohammad A. Kher-Elden, Ali Sadeghi, Zakaria M. Abd El-Fattah, Kewei Sun, Saika Izumi, Satoshi Minakata, Youhei Takeda, Jorge Lobo-Checa

**Affiliations:** † Research Center for Advanced Measurement and Characterization, National Institute for Materials Science, 1-2-1, Sengen, Tsukuba, Ibaraki 305-0047, Japan; ‡ Graduate School of Pure and Applied Sciences, University of Tsukuba, Tsukuba 305-8571, Japan; § Physics Department, Faculty of Science, Al-Azhar University, Nasr City, E-11884 Cairo, Egypt; ∥ Department of Physics, Shahid Beheshti University, 1983969411 Tehran, Iran; ⊥ School of Nano Science, Institute for Research in Fundamental Sciences (IPM), 19395-5531 Tehran, Iran; # Department of Applied Chemistry, Graduate School of Engineering, Osaka University, Yamadaoka 2-1, Suita, Osaka 565-0871, Japan; ∇ Instituto de Nanociencia y Materiales de Aragón (INMA), CSIC-Universidad de Zaragoza, E-50009 Zaragoza, Spain; ○ Departamento de Física de la Materia Condensada, Universidad de Zaragoza, E-50009 Zaragoza, Spain

**Keywords:** Surface state confinement, superatom molecular states, halogen-bonding network, scanning tunneling microscopy and spectroscopy, density functional theory calculations, electron plane wave expansion simulations

## Abstract

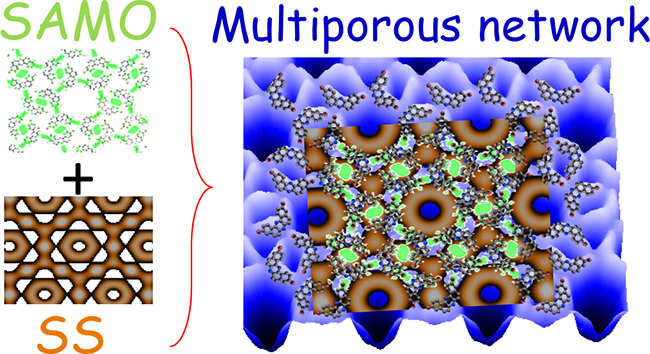

Two-dimensional honeycomb molecular networks confine a substrate’s surface electrons within their pores, providing an ideal playground to investigate the quantum electron scattering phenomena. Besides surface state confinement, laterally protruding organic states can collectively hybridize at the smallest pores into superatom molecular orbitals. Although both types of pore states could be simultaneously hosted within nanocavities, their coexistence and possible interaction are unexplored. Here, we show that these two types of pore states do coexist within the smallest nanocavities of a two-dimensional halogen-bonding multiporous network grown on Ag(111) studied using a combination of scanning tunneling microscopy and spectroscopy, density functional theory calculations, and electron plane wave expansion simulations. We find that superatom molecular orbitals undergo an important stabilization when hybridizing with the confined surface state, following the significant lowering of its free-standing energy. These findings provide further control over the surface electronic structure exerted by two-dimensional nanoporous systems.

Confinement of surface electrons within nanoporous molecular networks^1−5^ is a rapidly progressing field since the first demonstration of the quantum corrals fabricated by a series of atom-by-atom manipulations.^6−9^ The molecular self-assembly protocols, leading to zero-dimensional confining structures, provide a myriad of geometries that efficiently trap the surface state (SS) electrons from the substrate.^3,10−12^ Among these, halogen-bonded molecular networks have shown great versatility due to the high controllability of their bonding strength and direction.^13,14^ The mixed bonding character of aryl-halide groups^15,16^ facilitates the formation of unique two-dimensional (2D) porous structures, e.g., polymorphic,^17^ honeycomb,^14^ or Sierpinski triangle fractals.^18^

The constituents of the organic networks create regular potential barriers that efficiently scatter the surface electrons.^2,5,14,19−23^ The electron confinement is imperfect at nanocavities, so when nanoporous networks become extended over the metal surface quantum dot (QD) bands materialize.^4,5,14,22^ These modified bands exhibit energy dispersion of the otherwise flat local density of states (LDOS), evidencing the existence of electronic intercoupling between neighboring pores, whose magnitude is ultimately defined by the network building units (molecules and/or metals) .^5,14,22−24^

Different in kind to SS resonances, 2D organic networks can host molecular pore states at sufficiently small nanocavities, named as superatom molecular orbitals (SAMOs). These unoccuppied states feature high, site-dependent LDOS out-protruding from the carbon backbone into the pore that leads to molecular orbital (MO) overlapping with adjacent molecules.^25−28^ Notably, SAMO undergo significant energy downshifts with respect to their calculated free-standing values.^25,27^ Understanding the driving mechanism for such recurring observation becomes essential for designing MO-based devices with high, site-dependent LDOS (organic thin film transistors, organic light emitting devices, or photovoltaic cells)^27^ since their energies require proximity to Fermi to be exploitable. Moreover, such tiny pores are important in membranes as chemical and biological sensors.^25^ Thus, unraveling the unknown SAMO stabilization mechanism, which defines its energy, and its possible coexistence and interaction with SS resonances at the pore sites of a single organic network is essential.

In this work, we explore the electronic structure in a novel 2D halogen-bonded network containing three kinds of pores formed on the Ag(111) surface. Using a combination of low-temperature scanning tunneling microscopy and spectroscopy (STM/STS), density functional theory (DFT) calculations, and electron plane wave expansion (EPWE) simulations, we study in depth the pore states. We find that confined SS resonances exist within all nanocavities and coexist with at least one dominant SAMO state. The hybridization of these two kinds of pore states is responsible for the dramatic energy downshift of the free-standing MO defining the SAMO. Furthermore, text-book overbarrier resonances stemming from the largest pores coincide with the smaller pore sites, suggesting an influence of the modulated SS on the final network conformation.^23,29^ In consequence, the choice of the array’s molecular building blocks and the substrate turns out to be interdependent when searching for intense LDOS resonances on 2D organic films. From the molecular device perspective, this opens a novel engineering field intended for tuning these SAMO resonant energies by playing with the underlying substrates, which could be in the form of metallic monocrystals, thin films, or ultrathin surface alloys such as BiAg_2_ or GdAu_2_.

## Structure of the Organic Network: STM and DFT

**Figure 1:**
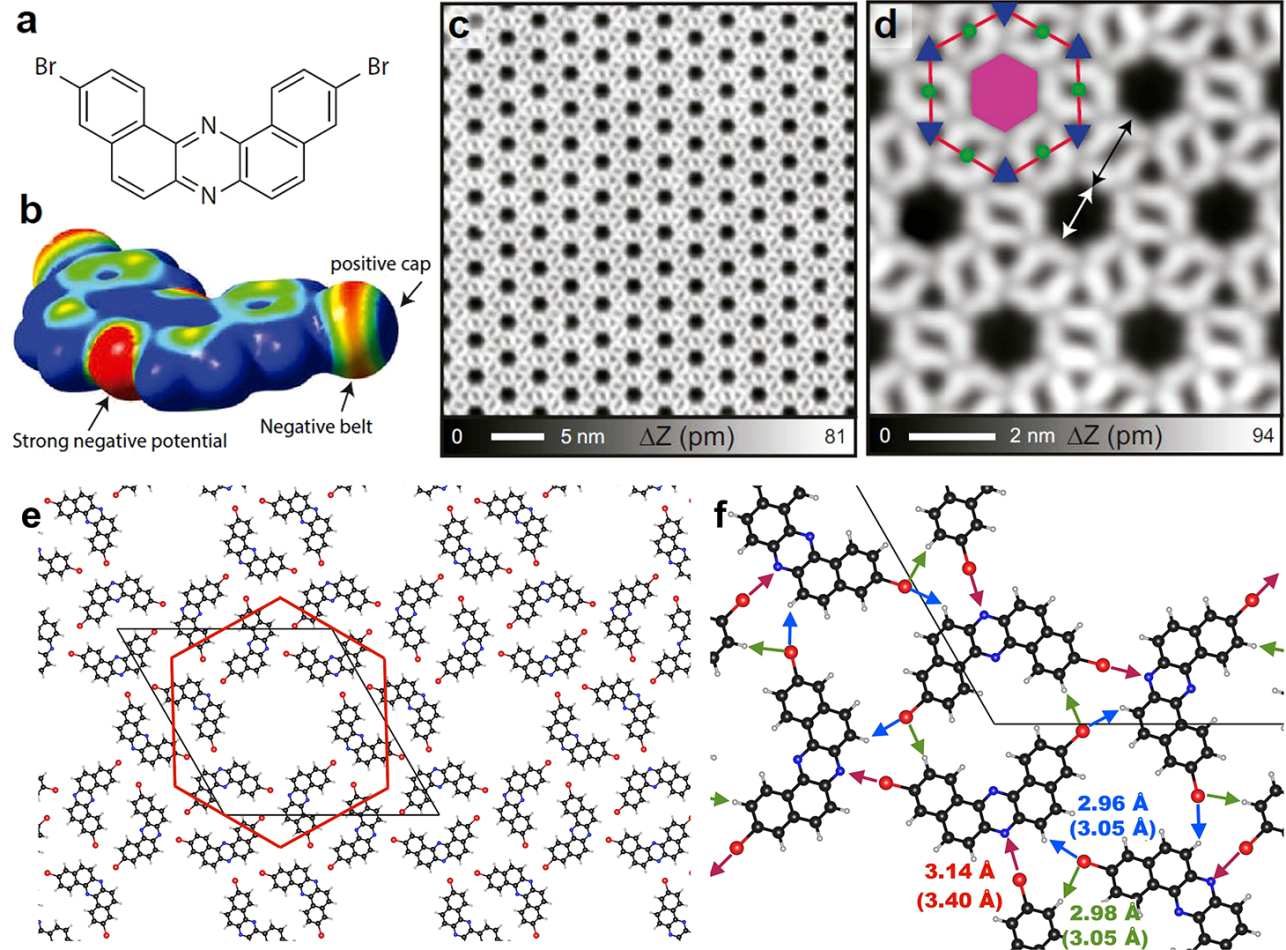
Molecule and self-assembled structure of the multiporous halogen-bonded network grown on Ag(111). (a) Molecular structure of 3,11-dibromo[*a*, *j*]phenazine consisting of two sets of bromo-substituted naphthalene moieties fused to a pyrazine core. (b) Electrostatic potential map for the iso-density surface (0.001 e/Bohr^3^) of the precursor obtained from DFT electrostatic potential calculations, displayed in a red–green–blue colorscale (±15 mV range). (c) Large-scale and (d) close-up view of STM topographies of the network. Three types of pores can be identified within the array that are named with respect to the red hexagon as central (magenta hexagon, area of ∼2.0 nm^2^), edge (green circles, area of ∼0.18 nm^2^), and corner (blue triangles, area of ∼0.21 nm^2^) pores. (e) DFT calculated hexagonal molecular network on Ag(111) marking the red hexagon and the primitive cell. (f) Close-up view indicating the intermolecular distances below 3.5 Å. The numbers in parentheses indicates the sum of the van der Waals radii. Each central pore is defined by six straight C–Br··· N halogen bonds (red arrows), while the edge (corner) pore is defined by two (three) hydrogen bonds [green (blue) arrows]. Measurement parameters: sample voltage *V* = 10 mV/tunneling current *I* = 100 pA in (c) and *V* = 10 mV/*I* = 10 pA in (d).

The complex, multiporuous, halogen-bonded network was obtained by the self-assembly of 3,11-dibromo[*a*, *j*]phenazine molecules^30^ (Figure 1a) on the Ag(111) surface. It is condensed through strong electrostatic interactions of the electronegative outer nitrogen at the pyrazine core with the positive cap on the bromine atom in an adjacent molecule (Figure 1b), rather than the usual homohalogen bonding.^14,31^ Likewise, the opposite C–Br end binds to two other molecules through their external hydrogens. The STM topographies (Figure 1c, d) show a highly periodic structure constituting three different nanocavities, which we name central (magenta hexagon), edge (green circle), and corner (blue triangles) pores based on their position with respect to the red hexagonal unit cell (Figure 1e). The central pore is largest with a diameter of ∼1.5 nm, which is smaller than the distance to its adjacent in kind (∼1.9 nm) (cf., white and black arrows in Figure 1d). The corner and edge pores are significantly smaller than the central pore. Note that two additional phases of nonmultiporous assemblies were also found (Figure S1)

To gain insight into the bonding nature of the molecular assembly on Ag(111), we performed DFT calculations (see Methods section). As found experimentally, the most favorable conformation is hexagonal with 3.28 nm cell edges that enclose six molecules sequentially rotated by 60° (Figure 1e and Figure S2). Each molecule develops six bonds to its five neighbors and participates in defining the three different pores (Figure 1f). The chiral, 6-fold symmetric conformation of the network around a central pore occurs by the six straight C–Br··· N halogen bonds (red arrows). It can be considered a moderate bond compared to previously reported molecular crystals (bond range = 2.82–2.98 Å),^32^ yet it still plays a decisive role in this self-assembly.

At the opposite molecular end, the other Br binds with two neighboring hydrogens. First, the negative belt of Br attracts the hydrogen atom and forms the H··· Br bond as indicated by a green arrow. Note that this bond is responsible for the dimer formation that host the edge pores (Figures S1e and S1f). Next, the positive cap points to another H atom (blue arrow), three of which define the corner pores. Our DFT calculations identify this second type of H··· Br bond as rather unstable, emerging as an unfavorable gas-phase trimer. This explains the experimental absence of plain corner-pore arrays, whereas networks with independent edge and central pores were identified (Figure S1). In fact, this unfavorable alignment of the second H··· Br bond entails a slight electrostatic repulsion that lifts the Br atom away from the surface. Such a tiny vertical distortion (of only 0.25 Å) allows the selective tip-induced debromination by applying sample voltages of 1.7 V or higher (Figure S3). Nevertheless, we regard this organic layer as practically planar with a distance of 2.96 Å to the Ag(111). The overall adsorption energy of this network is 3.01 eV, where 0.44 eV corresponds to the cohesive energy of the free-standing layer.

## LDOS at the Network Pores: Coexistence of SS Resonances and SAMO States

**Figure 2:**
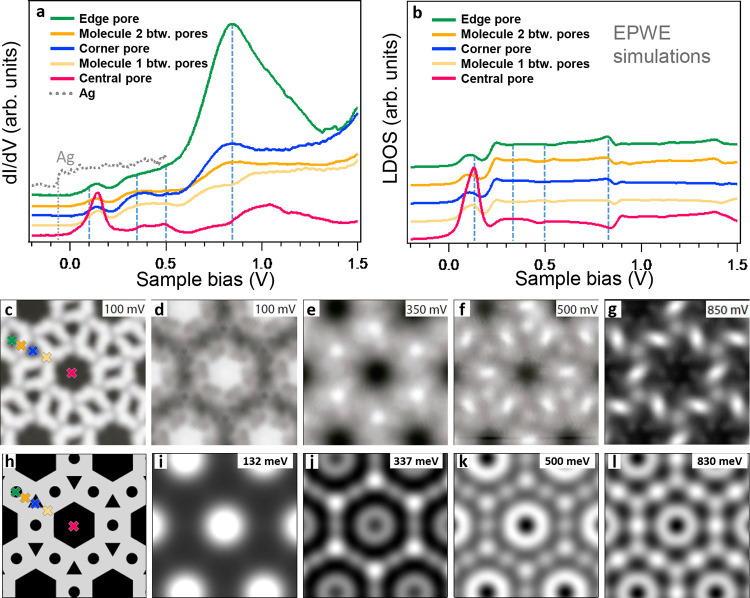
Measured LDOS of the network and corresponding EPWE simulation. (a) The d*I*/d*V* curves measured at the center of the three pore types and at neighboring molecules. The gray dotted line corresponds to the pristine Ag(111) reference, displaying the surface state onset at −68 mV. (b) LDOS spectra simulated by EPWE at the corresponding experimental sites. (c) STM topography of the multiporous network and the corresponding d*I*/d*V* maps at different energies in (d–g). (h–l) LDOS maps simulated by EPWE using a realistic geometry (see Figure S2). The crosses in (c) and (h) indicate the positions where the spectra shown in (a) and (b) are taken. Measurement parameters: *V* = 100 mV/*I* = 200 pA for (c); *I* = 200 pA in (d–g) with lock-in parameters *V*_rms_ = 7 mV. EPWE parameters: *V*_Ag_ = 0 mV (in black), *V*_molecules_ = 255 mV (in gray), *E*_SS_ = −70 meV, m* = 0.39 *m*_e_.

The LDOS data of the multiporous network were investigated by a series of site-dependent point STS measurements. We found that the STS lineshapes significantly differ from that of the Ag(111) substrate, which is practically featureless except for the SS onset at −68 mV (Figure 2a). To understand the nature of the observed STS peaks, we measured a series of differential conductance (d*I*/d*V*) maps at selected energies (Figure 2d–g and Figure S4), which provide spatial information on the DOS localization (Figure 2c).

The lowest energy peak of the network within the probed energy range was found at ∼132 mV. This state is mainly localized at the central pore in the d*I*/d*V* map acquired close to that energy (100 mV, Figure 2d). Its dome-like shape identifies it as the first confined SS resonance (*n*_central_ = 1). The large energy shift with respect to the SS onset (∼200 meV at the maximum) relates to the significant separation between adjacent central pores. Notably, this state leaks into the rest of the network as seen in all of the d*I*/d*V* curves (Figure 2a). Such ubiquity reflects its Bloch-wave nature that results in defined QD band structures.^4,5,14,22^ However, there is a rather limited intercoupling due to the large separation between adjacent pores (cf., narrow *n* = 1 peakwidth).

The next relevant STS peak maximizes at the corner pores around 340 mV and dominates the corresponding d*I*/d*V* map (Figure 2e). We attribute it to the first SS resonance of the corner pore (*n*_corner_ = 1), which coincides with higher resonances of the central pores (*n*_central_ = 2). Likewise, a localized LDOS at the edge pores was observed around 500 mV (Figure 2f), corresponding to its first SS resonance (*n*_edge_ = 1). Thus, all pores have SS resonances at different energies. In essence, the significant energy differences found at the *n* = 1 SS resonances (identified from their enhanced LDOS signal at the pore centers) relate to their enclosed areas, pore morphology, network symmetry, barrier potentials, and intercoupling. Furthermore, we found the LUMO onset in an energy around 200 and 250 meV in the d*I*/d*V* maps (Figure S4), coinciding with a weak intensity increase in the STS curves (Figure 2a.)

The dominant peak in STS appears at the edge pore (∼850 mV, green curve in Figure 2a), sharing a much weaker contribution at adjacent corner pores and being absent at the central pore (Figure 2g). This pore state is so pronounced that cannot be conceived to be the same kind as the SS resonances. Moreover, unlike the SS resonances that extend over the nanocavity contours, this one sharply reflects the chirality of its pore. Furthermore, such a dominant state (upshifted by 250 meV) can also be observed in the condensed assembly (Figure S5) that features edge pores only. Therefore, we tentatively assign it to be of molecular origin, i.e., a SAMO state.^25−27^

## DFT Identification of SAMO States

**Figure 3:**
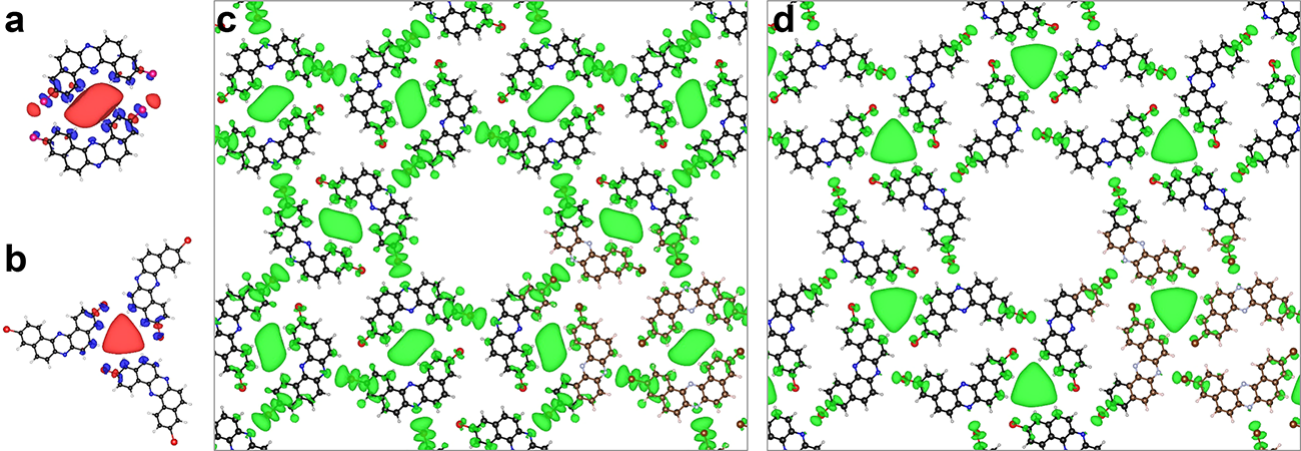
DFT hybrid orbital representation closely resembling the experimentally observed SAMOs. (a) Edge and (b) corner pore SAMOs of an isolated dimer and trimer, respectively. These evolve from the highly delocalized LUMO+8 orbitals of the monomers and reflect the pore chirality. Notably these are still preserved at certain energies in the free-standing network as hybrid SAMOs at edge (c) and corner (d) pores. The iso-surfaces correspond to an electron density of 2 × 10^–4^ a.u. and has contribution from a single orbital [17th, 25th, and 49th unoccupied orbitals of dimer (a), trimer (b), or network (d)] or a set of degenerate orbitals [52nd and 53rd network orbitals (c)] (see Figures S6 and S7).

To confirm the presence of SAMO states in our system, we performed DFT calculations and investigated the spatial shape of the unoccupied MOs in four free-standing cases, namely, a monomer, a dimer, a trimer, and the network (Figure S6). The monomer shows that most unoccupied MOs are highly localized on the carbon backbone or the end Br atoms, except for the ninth one (i.e., LUMO+8) that spreads out significantly. In a pore edge configuration, this state hybridizes between two adjacent molecules filling the dimer interspace (Figure 3a). The same result occurs for trimers in a corner pore geometry (Figure 3b). Thus, these SAMO states are feasible within the multiporous network and possess slightly different energies at corner and edge pores in the free-standing network configuration (Figure 3c, d). After aligning the LUMO to the experiment (∼220 meV), we find that the SAMO orbital of the edge pore must reduce its energy by ∼1.8 eV (Figure S7), which implies that a strong stabilizing mechanism in the form of an electronic hybridization must be at work. Note that smaller energy downshifts between DFT and experiments have already been reported for SAMOs.^25,27^

## Confinement SS Resonance Recognition Using EPWE Simulations

A candidate for such SAMO energy stabilization is the substrate itself. In the case of Ag(111), the electron density at the surface is dominated by the SS, which becomes strongly modulated by scattering with the organic network. The confined SS resonances at the pores can be identified from semiempirical EPWE simulations, which rely on the construction of a potential landscape recreating the molecular barriers of the nanoporous network, and then solves the associated electron plane wave scattering, which accounts for the quasi-free SS electrons (see Methods for details). When applied to nanoporous molecular networks, these simulations provide the real-space LDOS (containing the confined SS resonances) and quantify both the strength of the potential barriers and the dispersion of the QD array band structures.^1,2,14,22,23,33^ Since this modelization cannot consider the MOs, we take advantage of this limitation to discriminate the confined SS resonances from the SAMO states.

The network geometry for our EPWE simulations simplistically contains two potential regions (Figure 2h), namely, organic barriers (*V*_molecules_ = 255 mV in gray) and hollow regions (*V*_Ag_ = 0 in black). Using the Ag Shockley state as a reference (*E*_B_ = −0.07 eV and *m*_eff_ = 0.39 *m*_e_), we match the overall electrostatic potential map extracted from the DFT calculations (Figure S2). Our simulated LDOS spectra (Figure 2b) and the experimental data sets (Figure 2a) are only reliable up to 200 mV, coinciding with the upper molecular barrier energy (*E*_barrier_ = *E*_SS onset_ + *V*_molecules_ = −70 + 255 = 185 mV) and just below the LUMO onset. Above 200 mV, the absence of MOs in the simulations causes LDOS lineshapes to stray from the STS. However, an edge pore resonance at the 830 mV peak is visualized, although it is very weak in comparison.

Despite these considerable differences in the spectra, the simulated LDOS maps (Figure 2i–l) are surprisingly similar (disregarding chirality) to the experimental ones at the selected energies (Figure 2d–g). In particular, the *n* = 1 SS resonances of the smaller pores clearly appear at the corner pore (∼337 meV, Figure 2j) and weakly at the edge pore (500 meV, Figure 2k). Importantly, an energy coincidence at 830 mV is found between the SAMO state and the second confined SS resonances of the edge pore (Figures 2b and 2l). The same situation occurs for the compact network but with a remarkable 250 mV rigid shift (Figure S5c). Such coincidence supports the hybridization scenario between the SS resonances and the SAMO.

## Experimental Validation of SS Resonance and SAMO Entanglement

**Figure 4:**
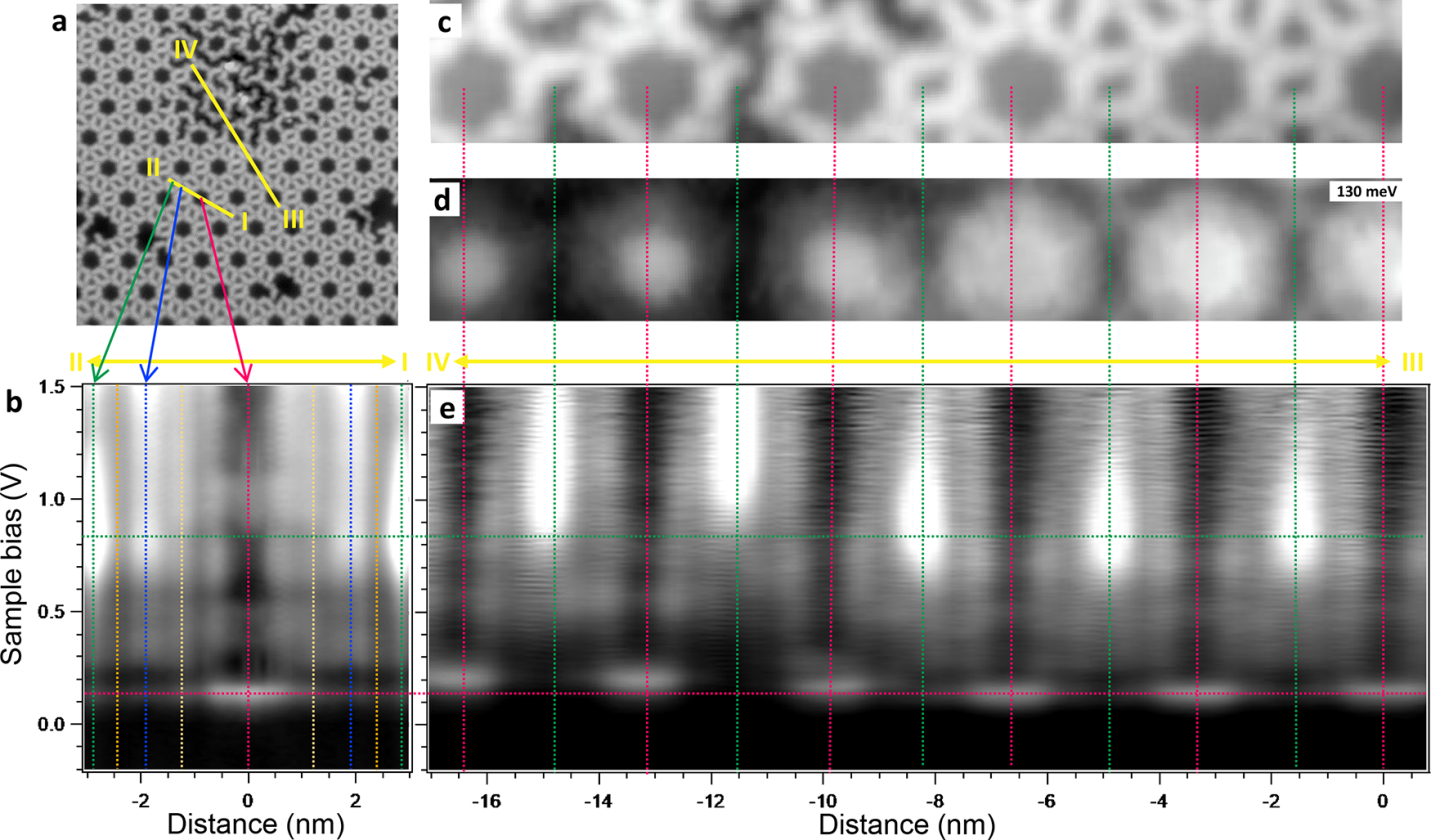
Linescans and defect-induced modifications upon the pore states. (a) STM topography of the network region defining the two linescans acquired (yellow lines). The defective region at the top was generated by selective tip-induced debromination (Figure S3). (b) Logarithmic grayscale d*I*/d*V* linescan along the direction connecting the three types of pores (I–II). The vertical lines mark the center of the pores and the molecular positions corresponding to Figure 2a. (c) Simultaneously obtained STM topography and (d) d*I*/d*V* map at the energy of the *n*_central_ = 1 SS resonance maximum that includes a defective area at the left part. (e) Logarithmic grayscale d*I*/d*V* linescan connecting the central and edge pores (marked with vertical lines) along the III–IV trajectory. The horizontal lines are a guide to the eye for the energy position of the network’s *n*_central_ = 1 SS resonance (red) and the SAMO state (green). Measurement parameters: *V* = −200 mV/*I* = 100 pA in (a); *V* = 130 mV/*I* = 200 pA in (c) and (d); the set point in (b) and (e) is *V* = −200 mV and *I* = 100 pA with lock-in parameter *V*_rms_ = 7 mV.

To verify the SS resonance and SAMO correlation, we performed two sets of d*I*/d*V* line-scanning over the network (Figure 4a). The first (I–II) connects the center of all pore types (Figure 4b). The intensity modulations match the above-discussed pore SS resonances, which are always practically quenched at the adjacent molecules. Interestingly, the corner pore (vertical blue line) explodes in intensity at the highest bias probed (upraising tail in the STS of Figure 2a and evident in Figure S4) marking the onset of the corner pore SAMO state predicted in DFT (Figure 3d).

The second d*I*/d*V* linescan (III–IV) joins six central pores and five edge pores (Figure 4c–e). In this case, selective debromination was conducted to introduce defects in the network (Figure S3) This maintains the integrity of the molecular backbone required for the LUMO+8 existence, but it alters the confining properties of the pores, as evidenced by the energy upshifts of the *n*_central_ = 1 resonance. Such energy shifts vary from pore to pore, affecting their electronic coupling with the rest of the network. Importantly, the SAMO states persist even in the presence of these defects. The fact that they exhibit similar energy shifts to the SS resonances after altering the network, independently of having one or two debrominated molecules defining the edge pore, experimentally validates their correlation with the confined SS resonances.

## Discussion

The combination of experimental results and two theoretical methods allows us to unambiguously identify two different types of coexisting pore states and to confirm their correlation within tiny pores of organic networks. The stabilization of the free-standing SAMOs results in a substantial energy downshift of almost 2 eV. Indeed, these edge-pore SAMOs exhibit the lowest experimental energy value reported to date (compare 0.8 V of this work with 1.8 V from ref (26), 2.1 V from ref (27), 2.2 V from ref (25), or unidentified SAMO states at 2.1 V in ref (34) or above 2.2 V in ref (35)). We deduce that the LDOS spatial distribution of the *n*_edge_ = 2 confined SS resonance promotes the LUMO+8 stabilization and hybridization as it creates a small ring overlapping with the molecular edges. Similarly, at the corner pores, the SAMO onset is in proximity to the *n*_corner_ = 4 SS resonance that also features LDOS maxima at the pore rims.

In this way, three properties become important for this SAMO-SS resonance correlation: (i) the size of the nanocavity, since the smaller it is the easier for the MOs to overlap and fill the void; (ii) the LDOS shape of the SS resonance that preferentially promotes the stabilization of SAMOs at the rims of the nanocavities; and (iii) the SS resonance energies participating in the hybridization with the SAMO state. The latter is experimentally confirmed in Figure 4 when the molecular debromination upshifts the SS resonances, thereby pulling the SAMO with them. The SAMO persists despite cleaving the C–Br bonds since the carbon backbone of the molecule is unaffected and the created radical at the Br position is electronically subdued through bonding with the underlying Ag substrate,^36^ which justifies the potential barrier modification around the molecule.

The EPWE simulations presented in Figure 2 should be compared to a solid geometry without corner and edge pores (Figures S8 and S9). In the extended, single large-hexagonal pore network, we find overbarrier resonances since the pore states can still interact and form Bloch waves modulated by the overall network potential landscape, i.e., despite the pores being so far apart, dispersive QD bands still form. Such overbarrier resonances are a textbook example materializing at precise symmetry dependent sites whenever *E* > *E*_barrier_. The largest pore concentrates most of the modulated SS, thereby imposing its symmetry and periodicity to the rest of the network. Given the central pore’s hexagonal symmetry, we find the overbarrier resonances at corner and edge sites (Figures S8P,Q). Thus, we do not discard a SS-mediated growth scenario^23,29^ in the formation of our complex network that would further stabilize the edge and corner mini-pores and compensate the relatively small dimerization energy found by DFT for the C–Br··· N halogen bonds. Likewise, the presence of edge and corner pores contributes with a minor energy reduction of the central pore SS resonance energy.

## Conclusion

In summary, by using halogen bond motifs we obtained a complex multiporous network that hosts simultaneously SS resonances and SAMO states at the array’s tiniest pores. By means of a combination of low-temperature STM/STS, DFT calculations, and EPWE simulations, we validated their correlation and showed that the SAMO stabilization takes place via electronic hybridization with the unoccupied SS resonances. This is experimentally corroborated through the introduction of defects (selective debromination) in the network that modifies both pore states. The reported substrate–network interplay results in a remarkable energy reduction of specific, out-protruding MOs that brings them very close to the Fermi level. Our fundamentally relevant findings shed light into key aspects of electron confinement by self-assembled nanoporous networks. Such found interdependence between SS resonances and SAMO states is key to reach full control of modified band structures stemming from two-dimensional electron gases.

## Supporting Information Available

Method section (STM/STS, DFT calculations, and EPWE simulations) and some supplementary text that is accompanied by nine supplementary figures and associated references (PDF)
