# Supplementary material for: Near Fermi Superatom State Stabilized by Surface State Resonances in a Multiporous Molecular Network
Source: Nano Lett. 2021 May 26;21(15):6456–62. doi: 10.1021/acs.nanolett.1c01200 (PMC8488955; doi:10.1021/acs.nanolett.1c01200)
Supplement: Supplementary file 1 — nl1c01200_si_001.pdf [file nl1c01200_si_001.pdf]

Supplementary information of: Near Fermi super-atom state stabilized by surface state resonances in a multi-porous molecular network

Shigeki Kawai, Mohammad A. Kher-Elden, Ali Sadeghi, Zakaria M. Abd El-Fattah, Kewei Sun, Saika Izumi, Satoshi Minakata, Youhei Takeda, and Jorge Lobo-Checa

The content of this supplementary Information file is the following:

- The Method section (STM/STS, DFT calculations and EPWE simulations)
- Supplementary text
- Supplementary figures
- Supplementary references

METHODS

STM/STS measurements. All experiment were performed with home-build STM/AFM system, operating at 4.3K under ultra-high vacuum conditions. A clean Ag(111) surface was in-situ prepared by repeated cycles of standard sputtering and annealing. A sharp W tip was obtained by the chemical etching and was then in-situ covered with Ag atoms by contacting to the sample surface.
3,11-dibromo[*a*,*j*]phenazine molecules [1] were deposited on Ag(111) kept at room temperature from crucible of Knudsen cell. The sample was biased while the tip was electronically grounded. The topographic images were taken in a constant current mode.

DFT calculations. The calculations were carried out within the semi-local density approximation of density functional theory (DFT). The most favorable conformations were found in calculations by exploring systematically the potential energy landscape of the model system. To reduce the computational demand for our large system (containing 688 atoms), we limit our search to a few reasonable initial trail geometries that best fit the high-resolution experimental images, and then relax the atoms by nullifying the forces act-ing on them. The DFT computations were performed using the Quantum ESPRESSO package [2]. Electron-ion Interactions are expressed by the projector augmented wave [3] potentials while the electronic exchange-correlation (XC) is treated within the generalized gradient approximation in the Perdew-Burke-Ernzerhof format [4]. The semi-local approxi-mation to XC poorly describes the dispersive effects. To compensate for this shortcoming, we employed the semi-empirical DFT-D3 scheme of Grimme [5] with the Becke-Johnson damping function [6]. A kinetic energy cutoff at 400 eV for the plane-wave expansion and a width of 0.05 eV for a Gaussian type smearing with a final extrapolation of total energy to zero temperature was used for solving the Kohn-Sham equation. Due to large size of the cell, only the G point of the k-space was sampled. Electronic and ionic relaxation criteria were set to 0.01 meV and 0.05 eV/Å, respectively. To get an approximate electrostatic potential felt by the SS electrons from the molecular layer, we performed calculations on a free-standing layer of the molecule. To investigate how the molecular orbitals contribute to the observed SAMOs, we considered the unoccupied molecular orbitals of the layer as well as those of the trimer, dimer and monomers of the molecule isolated from the layer. We

used the pseudopotentials from http://www.quantum-espresso.org.

EPWE simulations. The combined Plane Wave Expansion (PWE) and Electron Boundary Element Method (EBEM) have been developed by García de Abajo and represents a scalar variant of the electromagnetic PWE/BEM extensively used for solving Maxwell equations and optical response for arbitrary shapes. It is based on Green's functions for finite geometries and electron plane wave expansion for periodic systems. For the band structure calculations, the particle-in-a-box model is extended to infinite 2D systems by defining an elementary cell and using periodic boundary conditions. Within the PWE code, solutions of the Schrödinger equation are represented as a linear combination of plane waves and a satisfactory convergence was achieved with a basis set consisting of ~ 50 waves, as detailed in Ref. [7].
EPWE consideration of Br atoms. We found that Br atoms gives no significant improvement to the results of our EPWE simulations. This is a consequence of the relatively small surface fraction occupied by these atoms that consequently interact very weakly with the extended surface electron wavefunctions in stark contrast to the chemisorbed Br atoms dissociated by on-surface reaction. [8].
2DEG parameters for surface state confinement. The surface state parameters used to simulate the electron confinement are $E_{ref} = -0.070$ eV and $m^*_{eff} = 0.39 m_e$. These value match the pristine case, which is expected for purely organic networks that do not have metal adatom coordination [9, 10]. These parameters and the potential value of the molecules (V_{mol} = 255 meV) are used to obtain the best match of the complex network and the condensed network shown throughout this work.

SELF-ASSEMBLED ARRANGEMENTS BY 3,11-BR2DBPHZ

Aside from the complex network studied in the main text, deposition of the 3,11-Br2DBPHZ precursor results in another two assemblies: A simple quasi-hexagonal assembly with irregular pores and a condensed structure both based on N$\cdots$H-C hydrogen bonds (see Fig. S1) [11, 12]. Notably, the latter contains pores identical to the edge pores of the complex network, which allows us to study its electronic structure and confirm the correlation

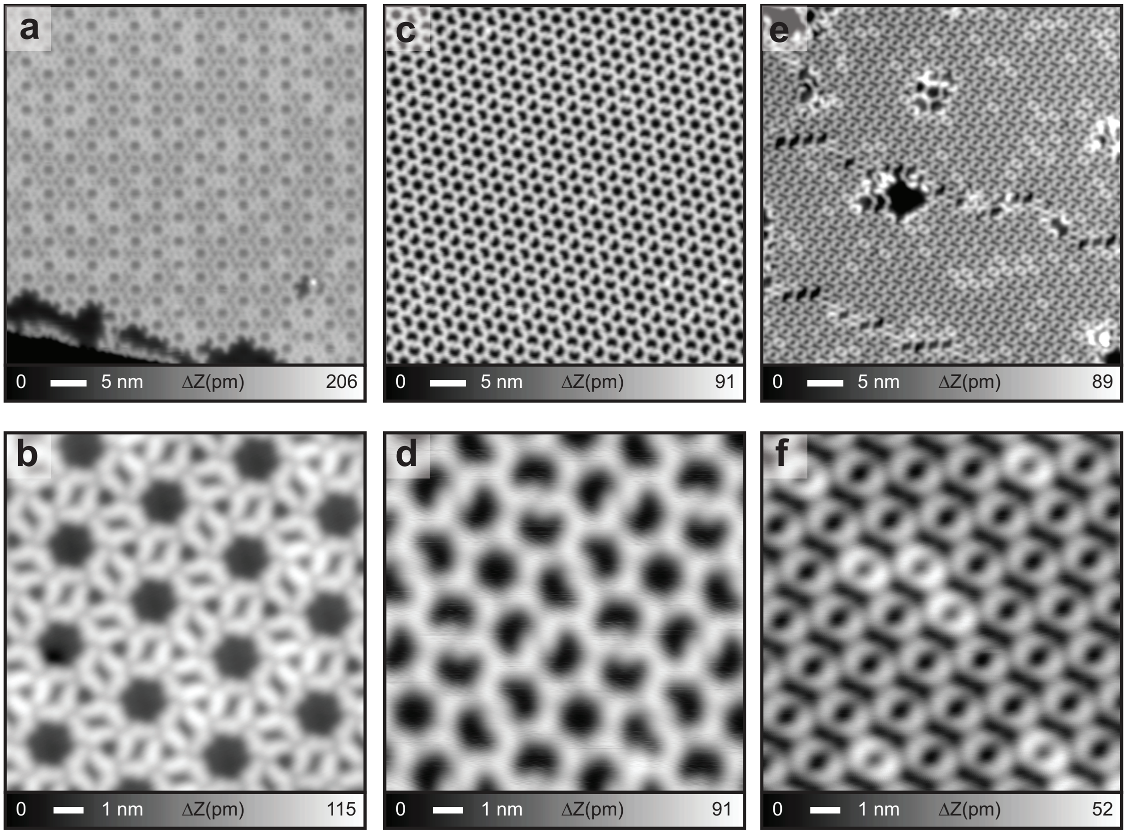

Figure S 1. Different arrangements observed by self-assembly of the molecules directly deposited on Ag(111). (a) and (b) shows the complex network studied in the main text, which dominates the surface when deposited on a substrate at room temperature. (c) and (d) shows a simple quasi-hexagonal assembly with irregular pores. (e) and (f) exhibits an assembly which we name as "condensed network" that is predominantly found when the deposition occurs with a cold substrate (slightly below room temperature). This condensed network consists of repeated edge pores units of the complex network (shown below) and visualized by the overimposed molecules. Measurement details: A,C,D,E,F: $V = 200$ mV / $I_S = 10$ pA; B: $V = 10$ mV / $I_S = 10$ pA.

between the confined SS resonances and the SAMO.

DFT DETAILS FOR THE COMPLEX HONEYCOMB STRUCTURE

In order to gain a deeper insight into the nature of the bonding of the complex hexagonal molecular network, we performed DFT calculations. To accurately determine geometric and energetic details of the inter-molecular bonding, appropriate corrections of dispersion effects

were introduced as described in Sec. Methods. It turned out that straight C-Br$\cdots$N halogen bonds are essential in formation of this molecular network. As illustrated in Fig. 1E, the conformation is a highly symmetric and chiral. An identical environment is seen around each molecule that is characterized by five molecules and one cavity (large spheres). Thus, the basis of the primitive cell of the hexagonal network consists of six molecules. This 3,11-Br2DBPHZ hexamer, looking like a regular hexagram, is a result of six C-Br$\cdots$N halogen bonds formed between six adjacent monomers as indicated by dashed ovals. Fig. 1F shows the closeup view around the corner of the primitive cell. As indicated by red arrows, the C-Br bond points to the outer N atom of the adjacent molecule. The halogen bonds are almost straight ($\angle$ C-Br$\cdots$N =178°) while the halogen bond length is 3.14 Å, i.e. 8 % shorter than the sum of the van der Waals radii of Br and N (3.40 Å), but it is stretched with respect to other (stronger) halogen bonds $2.82 - 2.98$ Å [13]. This bond is longer than a typical strong halogen bonds although the N atom in the pyrazine core shows a strong negative potential (Fig. 1B). We investigated the mechanism of this moderate halogen bonding. By relaxing the geometry of an isolated 3,11-Br2DBPHZ dimer condensed by a single halogen bond, we obtained a relatively low dimerization energy of 0.20 eV while the Br-N distance remains the same as in the network. In the honeycomb network, the belt of the bromine in the other side faces to the hydrogen atom in the adjacent molecule as indicated by green arrows (Fig. 1F). The contact is almost perpendicular ($\angle$C-Br$\cdots$H =106°) while the gap is 2.89 Å which is 5 % shorter than the sum of the van der Waals radii of Br and H (3.05 Å). Therefore, this contact can be categorized to a hydrogen bonding. We isolate one of the doubly H-bounded dimers from the network, and found that it is also stable (*albeit* a decrement of 7° is induced into the bond angle) and a dimerization energy of 0.19 eV is predicted. This bromine has another contact to the hydrogen atom in the adjacent molecule as indicated by yellow arrows (Fig. 1F). The contact with a length of 2.96 Å is an unfavorable bond because of $\angle$C-Br$\cdots$H = 167°, leading to the repulsion between H and the Br sigma hole. Indeed the C-Br$\cdots$H repulsion induces a great deformation during a geometry relaxation procedure in the network. In short, each molecule develops on average one set of halogen and hydrogen bonds as indicated by red and green arrows in figure 1F, respectively. When relaxed on the Ag(111) surface, the molecular network preserves the 6-fold rotational symmetry of the (111) surface using an 11×11 cell as the substrate. The layer remains almost planar at an average distance of 2.96 Å away from the plane of the metal topmost atomic layer, so it is considered

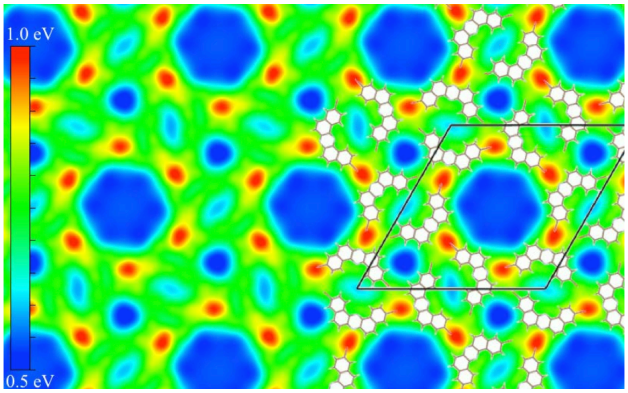

Figure S 2. Electrostatic potential map of the network obtained from DFT calculations. The electrostatic potential contour plot map is calculated at the last Ag surface layer (2.96 $\mathring{A}$ from the molecular backbone), where the surface state exhibits its maximum probability. According to this map, the electrons undergo a considerable repulsion at the C-Br$\cdots$N halogen bonds, whereas the C-Br$\cdots$H are relatively neutral in comparison. The blue colors of the three identified pores indicate the spatial regions where surface electron confinement exists.

physisorbed network. An outwards distortion of 0.25 Å is found in the Br atoms that are involved in the C-Br$\cdots$ H bonds. We attribute this distortion to the repulsion between H atom and the sigma hole of Br in the unfavorable connection previosly mentioned. The overall adsorption energy accounts to 3.01 eV/molecule, about 10 % of which is the cohesive energy of the hypothetical free standing layer.

SELECTIVE DEBROMINATION OF THE 3,11-BR2DBPHZ PRECURSORS

Our search for higher SAMO states resulted in the destruction of the network due to a de-halogenation process. Scanning the network with a bias voltage 1.7 V or higher resulted in a debromination from the 3,11-Br2DBPHZ precursors. Interestingly, this process is not occurring simultaneously at both Br ends of the precursor. As shown in Fig. S3, the end of the molecules that define the corner pores (the one with C-Br$\cdots$H bonding) are cleaved at this threshold bias. We infer that this is due to the upward distortion of these Br predicted by our DFT calculation that interact much more with the STM tip (and less with the substrate) and thus become easier to debrominate when charge is exchanged. We note that this selective process was used to generate the single pores within the network used in Fig. 4

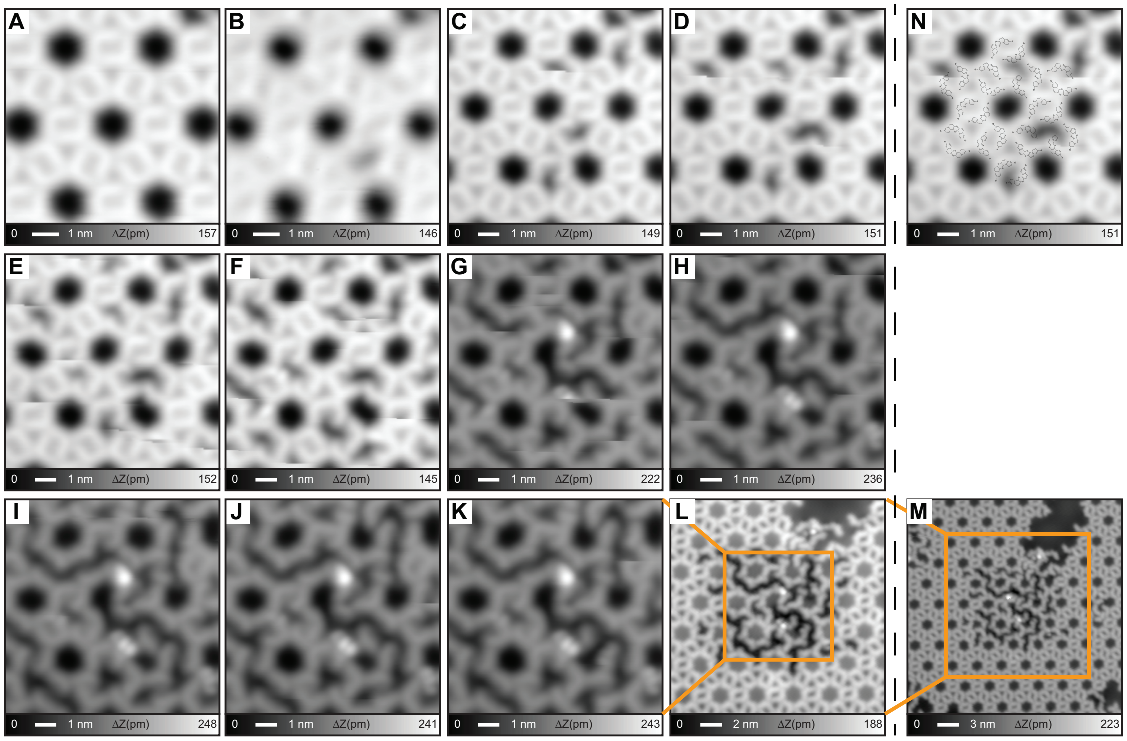

Figure S 3. Selective removal of Br atoms at the threshold voltage. (A) to (K) Sequential scanning of a particular region of the network using a 1.7 and 1.8 V bias demonstrating the selective debromination. At these voltages, the cleaving of the Brs happens only at the halogen ends with C-Br···H bonding. (L) and (M) shows two zoom-outs of (K), used to set the dI/dV linescans shown in Fig. 3 of the main manuscript. Panel (N) shows the overimposed molecular arrangement in (D), which clearly shows that the "broken" halogen ends correspond to the C-Br···H bonding. The STM parameters are shown below each image.

of the main manuscript.

EXPERIMENTAL dI/dV MAPS OF THE NETWORK

The selected experimental maps presented in Fig. 2 of the main text are extracted from Fig. S4. In these maps the evolution of the electron resonances can be easily followed. The central pore has its strongest resonance a low bias (around 100 mV) and then its intensity becomes relatively small with respects to other sites of the network. The molecules light up at 250 mV, marking the proximity of the LUMO onset energy. Around 350 mV it is the corner pores the ones that stand out and at 500 mV the edge pores begin to resonate.

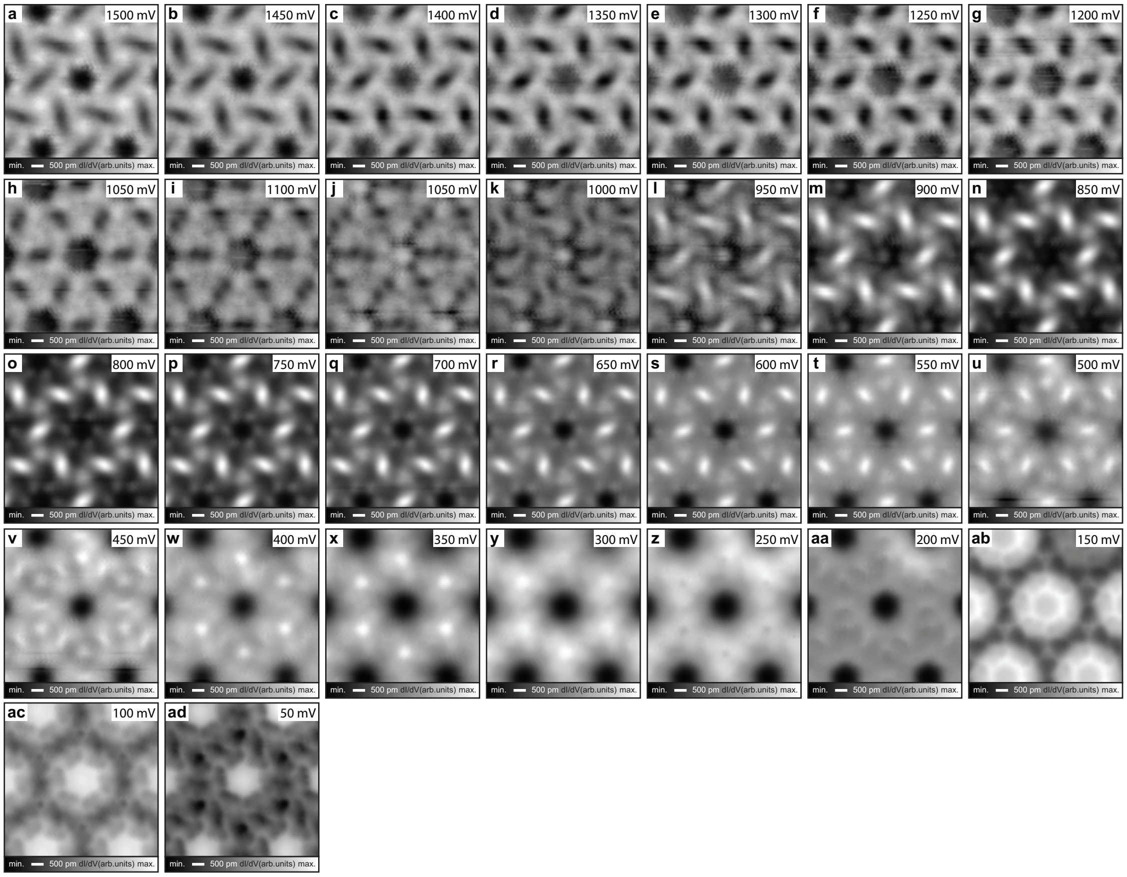

Figure S 4. dI/dV maps of the network acquired every 50 mV in the range from 50 mV (bottom) to 1500 mV (top). The features discussed in the main text, i.e., the confinement from the central and corner pores as well as the SAMO state on the edge pore are clearly observed. Moreover, at the highest energy acquired, there seems to be an emerging SAMO state at the corner pore, as discussed in the text. STS Parameters: The Voltage at which each map was acquired is shown on the top of each image, $I_S = 100$ pA; lock-in: $f = 510$ Hz, $V_{rms} = 10$ mV.

However, increasing the bias to ~ 800 mV results in the spectral domination of the SAMO that surpasses in intensity the rest of the network and shows the largest overall contrast. The next remarkable feature happens at the corner pore when reaching the highest energy proved (1500 mV), where we detect that the corner pore SAMO state onset is reached. However, we cannot go further in energy due to the start of the debromination process in the molecules, shown in the previous section.

ELECTRONIC STRUCTURE OF THE CONDENSED NETWORK

The correlation between the SAMO state and the confined surface state can be confirmed by comparing the electronic structure of the complex network in Fig. 2 of the main manuscript with the one of the compact network (shown in Fig. S5). This compact self-assembly dominates whenever the molecular deposition is done on a slightly cold substrate (below room temperature). Importantly, the compact network contains pores formed by two molecules that are identical to the edge pores in the complex network (cf. Fig. S1). However, experimentally the STS at this edge pore (green curve in Fig. S5C) differs from the one in Fig. 2A of the main text in the energy position of the SAMO resonance: in the complex network the SAMO was found at 850 mV, whereas in this compact network it is located at 1100 mV. Being the molecular dimer geometrically identical, the reason for this 250 mV shift cannot be related to the molecular states alone. Since the overall geometrical arrangement of the two networks is different, the modifications are induced by the energy position of the SS resonances. Indeed, the EPWE simulations in Fig. S5C shows at the pore site significant differences with respect to the complex network. These differences will be better visualized in Fig. S9. We find that the main difference stems in the energy position of the $n = 2$ resonance, which in the case of the complex network is found at 0.83 V, whereas in the compact network peaks at 1.25 V. This substantial difference is behind the observed shift of the SAMO due to the expected hybridization between the SS and SAMO.

DFT CALCULATED FREE-STANDING MOLECULAR ORBITALS

The unoccupied free-standing molecular orbitals (MO) have been calculated for four cases: a monomer, a dimer, a trimer and the complex network and are shown in Fig. S6. In the monomer, most of the MOs are highly localized on its carbon backbone, except for the nearly degenerate states LUMO+4 and LUMO+5 and the highly spread LUMO+8. However, in the dimer case, which contains the geometry of an edge pore, the MO overlap at the center is only significant for the LUMO+8. Indeed, when the network is calculated, this state is the one that exhibits the closest resemblance to the experimental SAMO found in the dI/dV maps of Fig. 2 and Fig. S4.

Once the MO responsible for the SAMO state has been identified, we represent in Fig. S7

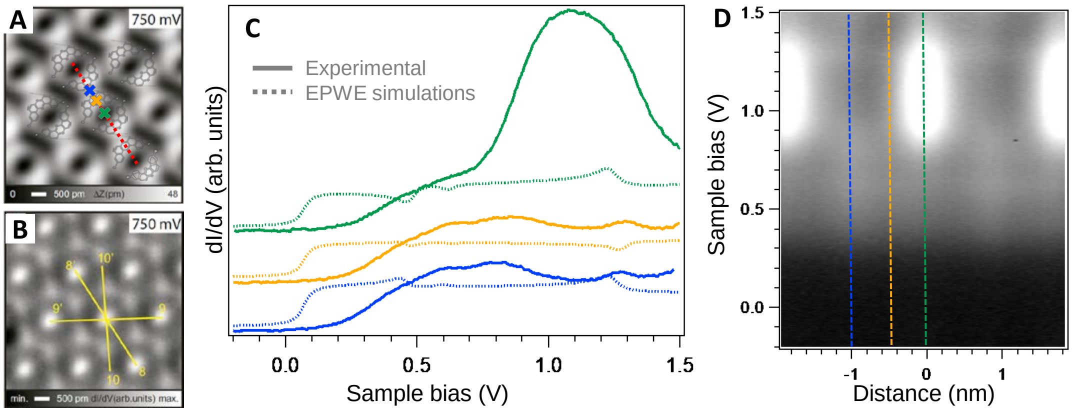

Figure S 5. Electronic structure of the compact network. The topography is show in (A) and a simultaneous dI/dV map at V_{bias} = 750 mV ($I_{Setpoint}$ = 100 pA) is shown in (B), with the brightest intensity located at the pores. (C) STS spectra at three selected positions (pore, molecule and midway) indicated by colored crosses on the red dotted line in A. The spectra displayed with the colored discontinued lines correspond to equivalent simulated spectra obtained by EPWE. (D) Experimental dI/dV linescan displayed in logarithmic grayscale and measured along the red line connecting adjacent pores that is marked in (A). The SAMO is located at the pores at 1.1 V.

their relative energies in a free-standing configuration. Aside from the known limitations of DFT regarding the energy position of the frontier orbitals, a significant energy downshift of these orbitals is to be expected just by the substrate presence. However, these are further downshifted whenever hybridizations of extended network orbitals are at play, which is the case here with our SS resonances. In particular, the LUMO is downshifted about 1 eV due to the substrate presence, whereas the initial LUMO+8 sees its energy reduction largely magnified due to the hybridization with the SS. We note that the LUMO+1 and LUMO+2 could also be present in the experimental energy window probed, but the strength of the SAMO state hinders from its proper identification.

EPWE SIMULATIONS OF THE COMPLEX AND COMPACT NETWORKS

The EPWE simulations calculates the surface state electron LDOS when they scatter against the potential barriers generated by the molecules of the network. It is important to stress that these simulations do not take into account any molecular states, so they cannot

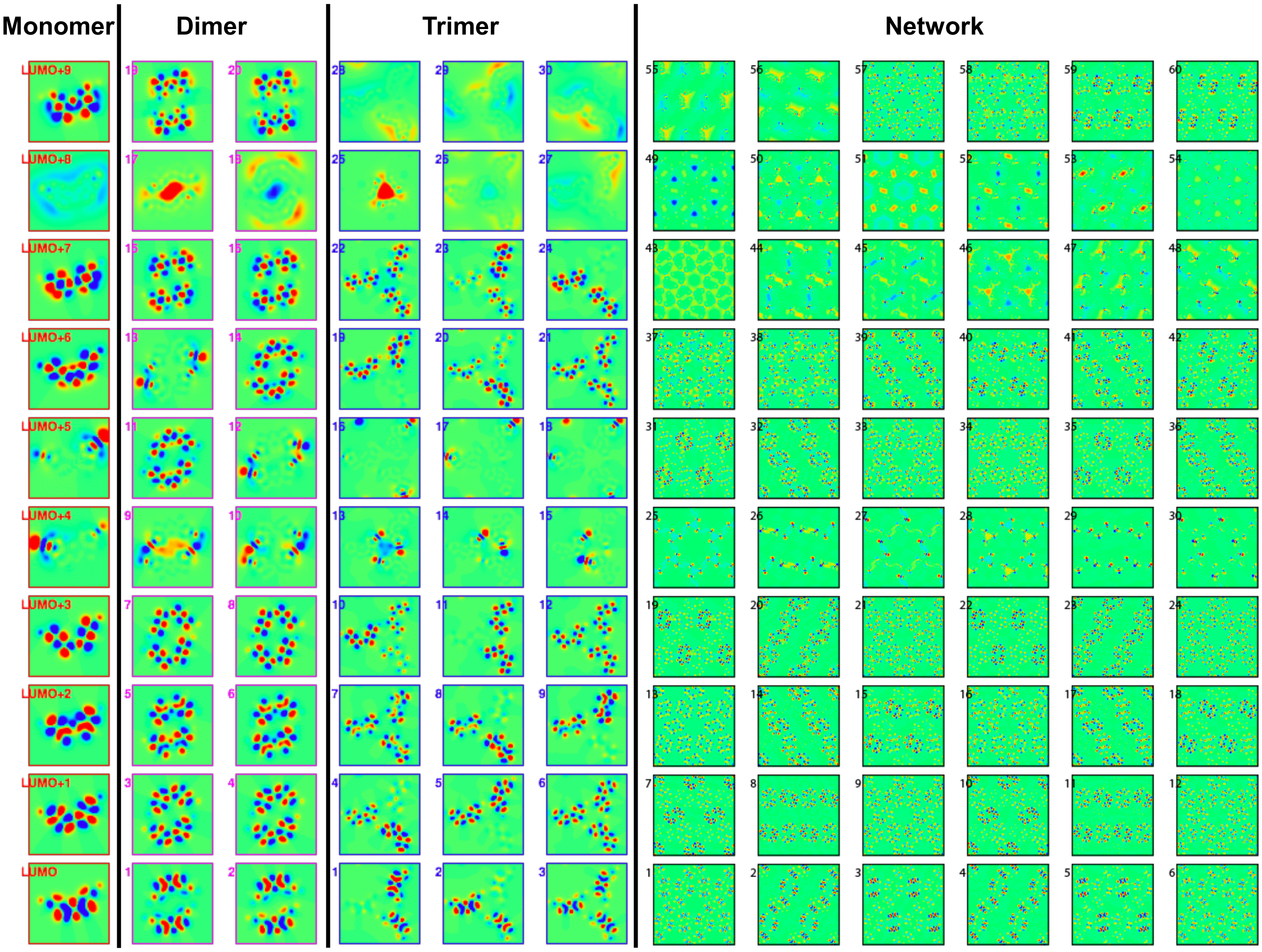

Figure S 6. DFT calculation of the unoccupied molecular orbitals arranged into monomers, dimers, trimers and the complex halogen network. The images represent the unoccupied molecular or hybrid orbitals, $|\Psi_n|^2 sgn(\Psi_n)$ where Ψ_n denotes the n-the unoccupied Kohn-Sham eigenfunction (orbital). The maps are the projection on a plane $1\mathring{A}$ above the molecular backbone plane in free-standing conditions (no underlying metal surface was considered). In each set, the panels are sorted and labeled by numerical indexes according to the orbital energies.

predict the SAMO state or any kind of modification to the molecular orbitals.

The methodology for the EPWE simulations has been described previously [9, 10, 14]. Being EPWE a semiempirical method, several starting assumptions based on the experimental STM/STS must be made in order to set the potential barrier strength and the molecular geometries. We start by modeling the networks (*i.e.* lattice constant and symmetry and

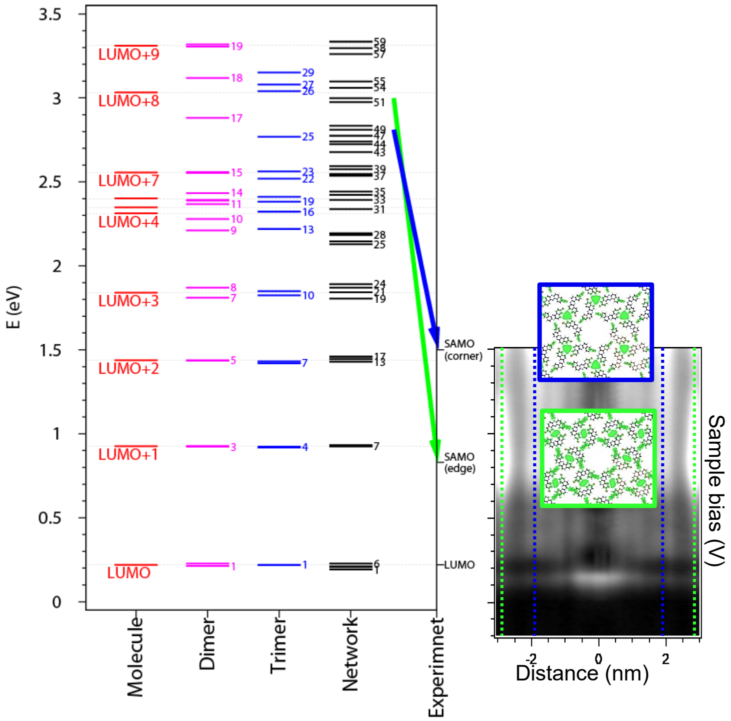

Figure S 7. Energy level diagram for the first ten unoccupied molecular orbitals of a free-standing molecule, dimer, trimer and a unit cell of the hexagonal network and comparison to the network in the experiment. In each case, the energy of LUMO is aligned to the value obtained in the experiment (Fig. 2 and 4 of the main text) and is also indicated on the right vertical axis for convenience. Tiny numerical labels indicate the orbital order as sorted according to their corresponding energies that relate to Fig. S6. The arrows show the important energy reduction that the extended LUMO+8 orbital undergoes when hybridizing with the SS resonance. On the right, we repeat the logarithmic grayscale dI/dV linescan along the direction connecting the three types of pores in Fig. 4 of the main text displayed on the same energy scale. The edge (corner) pore is marked by the green (blue) vertical line.

barrier widths) using our STM images and refined by chemical structures. Once the network geometry is fixed (with 3.4 nm periodicity), the surface state parameters (fundamental energy $E_0 = -70$ mV and effective mass $m^* = 0.39\ m_0$) are included and the scattering potential barriers is varied until the experimental STS and LDOS are reproduced. In our case we required to use for both networks $V_{mol} = 0.255$ eV. Note that since these networks are not metal coordinated we did not require to renormalize the SS [9, 10, 14]. Moreover, we purposely exclude the extra contribution from the Br atoms in the EPWE potential

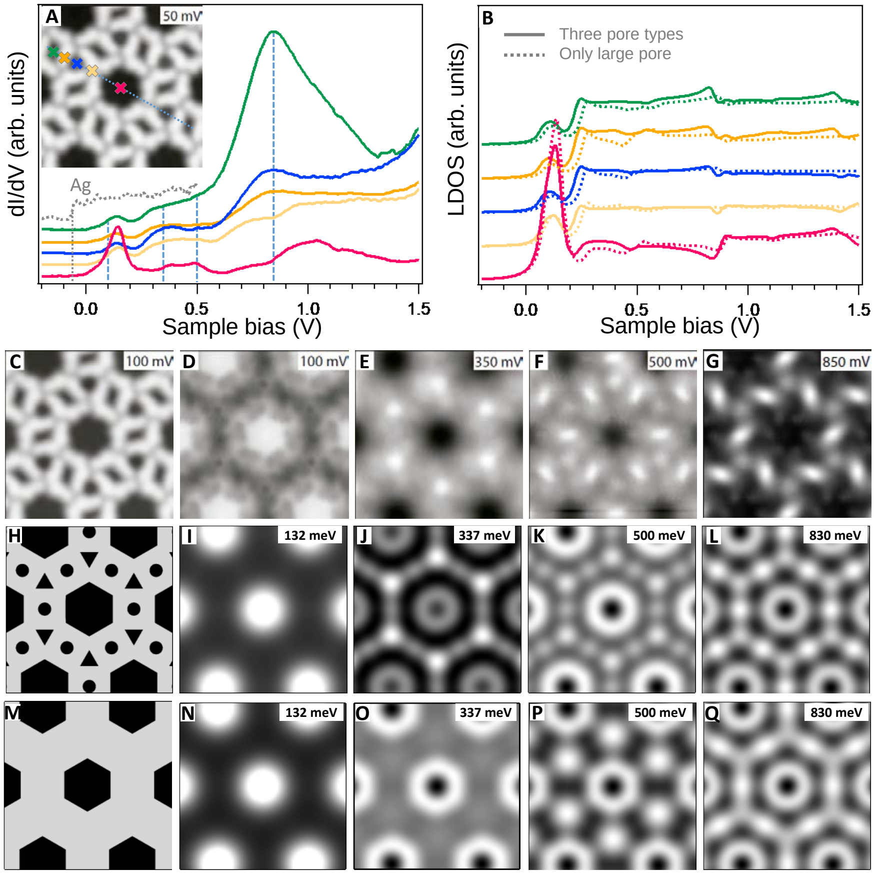

Figure S 8. Experimental LDOS of the network and two different EPWE simulations accounting for the SS resonances. The Figure contains the information displayed in Fig. 2 of the main manuscript but adds in the bottom row the simulation that considers only the central pores (black hexagons) separated by solid scattering walls (in gray). Although these LDOS maps (N to Q) have a very limited resemblance with the upper experimental ones, overbarrier resonances clearly coincide with the edge and pore positions, suggesting an influence of the SS in the final network geometry. Measurement parameters: $V = 100$ mV / $I = 200$ pA for inset of (A); $V = 10$ mV / $I = 10$ pA for (C); $I = 200$ pA for the different voltages in (D-G) with lock-in parameters $V_{rms} = 10$ mV. EPWE parameters: $V_{Ag} = 0$ mV (in black), $V_{molecules} = 255$ mV (in gray), $E_{SS} = -70$ meV, $m^* = 0.39\ m_e$.

geometries, as they did not bring any significant improvement in our simulations.

Fig. S8 displays the same information than Fig. 2 of the main manuscript, but an additional geometry consisting of only the largest pore and filling the space with a homogeneous barrier is considered in the bottom row. It is evident that there is a very limited resemblance

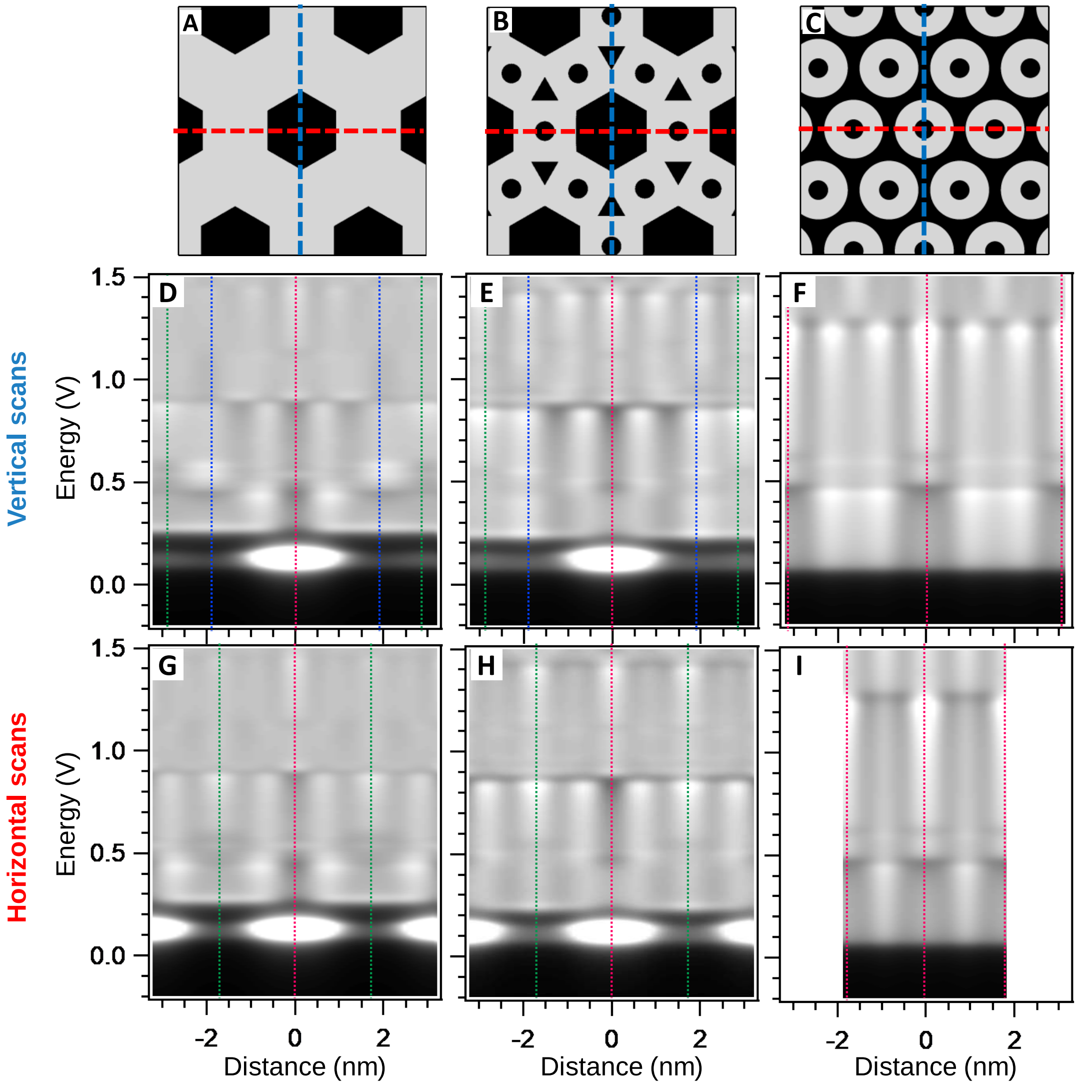

Figure S 9. EPWE simulations for three considered networks with the following geometries: (A) the complex network without the corner and edge pores, (B) the complex network and (C) the compact network. The gray area correspond to the molecular regions with the assigned scattering potential of $V_{mol} = 0.255$ eV. Contrarily, the black parts are zero potential regions where the underlying substrate is left uncovered. Below these geometrical models different 1D-LDOS grayplot maps are shown that run along the vertical (blue) lines [(D) to (F)] and along the horizontal (red) lines [(G) to (I)]. Although identical potentials have been imposed upon the two regions (gray and black) strong differences are observed in these plots, even in the very similar A and B cases. Overbarrier resonances are consistently found in the three networks. Note that these simulations only represent the surface state local intensity after being scattered by the network created potential barriers, but do not take into consideration any molecular state.

with the experimental datasets. However, for this network the existence of high energy over-barrier resonances (above the V_{mol} energy) close in energy and coinciding in position with the corner and edge pores suggest that the surface state influences the assembly and could stabilize the arrangement thereby mediating in the ultimate network geometry.

The final figure (Fig. S9) compares the simulated LDOS along two orthogonal high-symmetry lines of the three studied networks. In these 1D-LDOS grayscale plots, we observed the confinement resonances as brighter intensity. The position and energy dependent features are different for all networks, even for the very similar (A) and (B) networks, despite setting identical potentials in the black and gray regions. This shows that the SS resonances are highly dependent on the network geometry, which then conditions the final energy of the SAMO resonances. Comparing the edge pore of panels H with the central one in graph I of Fig. S9 we clearly observe the 250 mV shift of the top of the $n = 2$ resonance that we identify as responsible for the SAMO stabilization.

---

[1] Y. Takeda, M. Okazaki, and S. Minakata, Chem. Commun. **50**, 10291 (2014).

[2] P. Giannozzi, O. Andreussi, T. Brumme, O. Bunau, M. B. Nardelli, M. Calandra, R. Car, C. Cavazzoni, D. Ceresoli, M. Cococcioni, N. Colonna, I. Carnimeo, A. D. Corso, S. de Gironcoli, P. Delugas, R. A. D. Jr, A. Ferretti, A. Floris, G. Fratesi, G. Fugallo, R. Gebauer, U. Gerstmann, F. Giustino, T. Gorni, J. Jia, M. Kawamura, H.-Y. Ko, A. Kokalj, E. Küçükbenli, M. Lazzeri, M. Marsili, N. Marzari, F. Mauri, N. L. Nguyen, H.-V. Nguyen, A. O. de-la Roza, L. Paulatto, S. Poncé, D. Rocca, R. Sabatini, B. Santra, M. Schlipf, A. P. Seitsonen, A. Smogunov, I. Timrov, T. Thonhauser, P. Umari, N. Vast, X. Wu, and S. Baroni, J. Phys.: Cond. Mat. **29**, 465901 (2017).

[3] P. E. Blöchl, Phys. Rev. B **50**, 17953 (1994).

[4] J. P. Perdew, K. Burke, and M. Ernzerhof, Phys. Rev. Lett. **77**, 3865 (1996).

[5] S. Grimme, J. Antony, S. Ehrlich, and H. Krieg, J. Chem. Phys. **132**, 154104 (2010).

[6] A. D. Becke and E. R. Johnson, J. Chem. Phys. **127**, 154108 (2007).

[7] Z. M. Abd El-Fattah, M. A. Kher-Elden, I. Piquero-Zulaica, F. J. G. de Abajo, and J. E. Ortega, Phys. Rev. B **99**, 115443 (2019).

[8] M. Chen, J. Xiao, H.-P. Steinrück, S. Wang, W. Wang, N. Lin, W. Hieringer, and J. M.

Gottfried, J. Phys. Chem. C **118**, 6820 (2014).

[9] I. Piquero-Zulaica, A. Sadeghi, M. Kherelden, M. Hua, J. Liu, G. Kuang, L. Yan, J. E. Ortega, Z. M. A. El-Fattah, N. L. Behnam Azizi, and J. Lobo-Checa, Phys. Rev. Lett. **123**, 266805 (2019).

[10] I. Piquero-Zulaica, J. Li, Z. M. Abd El-Fattah, L. Solianyk, I. Gallardo, L. Monjas, A. K. H. Hirsch, A. Arnau, J. E. Ortega, M. Stöhr, and J. Lobo-Checa, Nanoscale **11**, 23132 (2019).

[11] S. Kawai, A. Sadeghi, F. Xu, L. Peng, A. Orita, J. Otera, S. Goedecker, and E. Meyer, ACS Nano **9**, 2574 (2015).

[12] I. Piquero-Zulaica, J. Lobo-Checa, A. Sadeghi, Z. M. A. El-Fattah, C. Mitsui, T. Okamoto, R. Pawlak, T. Meier, A. Arnau, J. E. Ortega, J. Takeya, S. Goedecker, E. Meyer, and S. Kawai, Nat. Commun. **8**, 787 (2017).

[13] A. Forni, The Journal of Physical Chemistry A **113**, 3403 (2009).

[14] I. Piquero-Zulaica, Z. M. Abd El-Fattah, O. Popova, S. Kawai, S. Nowakowska, M. Matena, M. Enache, M. Stöhr, A. Tejeda, A. Taleb, E. Meyer, J. E. Ortega, L. H. Gade, T. A. Jung, and J. Lobo-Checa, New Journal of Physics **21**, 053004 (2019).
